# Cognitive status and associated risk factors in high-risk older adults of China: a population-based cross-sectional study

**DOI:** 10.1186/s12877-026-07412-y

**Published:** 2026-04-01

**Authors:** Jindong Ding Petersen, Feng Chen, Kun Zhang, Yong You, Huijun Wei, Liangyan Yuan, Peng Lu, Bingbing Zheng, Chen Liu, Zhanjun Zhang, Jens Søndergaard, Frans Boch Waldorff, Wenting Cao, He Li, Tao Liu

**Affiliations:** 1 School of Public Health, Key Laboratory of Tropical Translational Medicine of Ministry of Education, Hainan Medical University, Haikou, China; 2 Department of Neurology, The Second Affiliated Hospital of Hainan Medical University, Haikou, China; 3 Section of General Practice, Research Unit for General Practice, Department of Public Health, University of Copenhagen, Copenhagen, Denmark; 4 Research Unit for General Practice, Department of Public Health, University of Southern Denmark, Odense, Denmark; 5 Department of Radiology, Hainan Affiliated Hospital of Hainan Medical University (Hainan General Hospital), Haikou, China; 6 Geriatric Center, Hainan Affiliated Hospital of Hainan Medical University (Hainan General Hospital), Haikou, China; 7 State Key Laboratory of Cognitive Neuroscience and Learning, Faculty of Psychology, Beijing Normal University; Beijing Aging Brain Rejuvenation Initiative (BABRI) Centre, Beijing Normal University, Beijing, China; 8 Beijing Aging Brain Rejuvenation Initiative (BABRI) Centre, Beijing Normal University; Institute of Basic Research in Clinical Medicine, China Academy of Chinese Medical Sciences, Beijing, China; 9 Department of Neurology, Hainan Affiliated Hospital of Hainan Medical University (Hainan General Hospital), Haikou, China

**Keywords:** Cognitive impairment, Mild cognitive impairment, Older adults, Population-based, Prevalence

## Abstract

**Background:**

Older adults at high-risk conditions are particular vulnerable to cognitive impairment; however, population-based assessments targeting this group remain limited. To exam cognitive status and associated risk factors in high-risk older adults in China.

**Methods:**

This population-based cross-sectional study was conducted across Hainan Province comprising adults aged ≥ 60 years with at least one high-risk condition: physical activity limitations, disability, stroke history, mental health symptoms, or subjective memory concerns, through stratified regional sampling. Pre-mild cognitive impairment (Pre-MCI) and MCI were assessed using the validated BABRI-brain health system. Demographic and health-related data collected from registries and self-reports. The prevalence rates of Pre-MCI and MCI were calculated, and multivariable logistic regression was applied to identify risk factors.

**Results:**

Among 228,087 participants (mean age 73.6 ± 8.6 years; 57.6% female; 67.1% with primary education or less), the prevalence of Pre-MCI and MCI was 51.4% and 32.0%, respectively. Significant risk factors for cognitive outcomes included older age (OR range for Pre-MCI: 1.27 [1.24–1.30] to 3.09 [2.77–3.45]; for MCI: 1.04 [1.01–1.07] to 4.28 [3.84–4.78]), unmarried status (OR 1.07 [1.03–1.12] for Pre-MCI; 1.45 [1.39–1.52] for MCI), lower education (OR range for Pre-MCI: 1.37 [1.32–1.41] to 3.74 [3.59–3.89]; for MCI: 1.61 [1.54–1.68] to 6.92 [6.60–7.25]), occupation as farmer/housemaker (OR 1.37 [1.31–1.43] for Pre-MCI; 1.59 [1.51–1.67] for MCI), and residence in medium GDP regions (OR 1.74 [1.66–1.82] for Pre-MCI; 1.38 [1.32–1.45] for MCI). Additional risk factors included hearing impairment, cerebral hemorrhage, family dementia history, and hypertension. Sex-specific differences were observed.

**Conclusion:**

The high prevalence of cognitive impairment in high-risk older adults highlights the need for tailored public health strategies. Particular attention should be given to those who are unmarried, with lower education, hearing impairment, and hypertension.

**Supplementary Information:**

The online version contains supplementary material available at 10.1186/s12877-026-07412-y.

## Background

Dementia is a global public health challenge, currently affecting an estimated 57 million individuals worldwide, with projections indicating an increase to 153 million by 2050 [1]. This escalating prevalence is reflected in China, where approximately 15 million people are currently living with dementia, a figure expected to rise to 40 million by 2050, imposing substantial socio-economic and public health burdens [2].

Mild cognitive impairment (MCI), an intermediate stage between normal cognitive function and dementia, is prevalent among community-dwelling older adults, with the prevalence ranges from 5.1% to 41.0% (median: 19.0%), and higher rates observed among older populations and individuals at high risk [3, 4]. In China, a 2018 study estimated the national prevalence of MCI at 22.2% among adults ≥ 60 years, with notable regional differences ranging from 29.9% in the southwest to 16.5% in the north [5]. More recently, a recent meta-analysis involving 393,525 adults ≥ 40 years reported an overall MCI prevalence of 19.6% [6]. Notably, over 40% of individuals with MCI progress to dementia within 5 years [3].

The cognitive status and associated risk factors in high-risk older adults may differ from those of the general population. High-risk groups, including individuals with disabilities, stroke history, or mental health conditions, exhibit significantly higher rates of cognitive impairment [7–9]. However, systematic studies examining these populations in China remained limited.

Recent studies have identified multiple determinants influencing cognitive function in high-risk populations, spanning biological, psychological, and environmental domains. Modifiable risk factors account for approximately 40% of dementia cases globally [10], while stroke survivors facing a 70% higher risk of developing dementia [11]. Depression increases dementia risk even further, with a 73% through mechanisms involving neuroplasticity impairment [12, 13].

Lifestyle factors also play a critical role. Physical inactivity, poor diet habits, and sleep disturbances worsen cognitive decline, whereas regular exercise and adherence to a Mediterranean diet have shown to exert protective effects [14]. Social and environmental factors, including low socioeconomic status, limited education, and social isolation, are also associated with higher rates of cognitive impairment [15], conversely, social engagement and cognitive stimulation have been linked with a reduced risk [16]. Disabilities and functional impairments, particularly those affecting mobility or activities of daily living (ADLs), exacerbate cognitive decline by reducing physical activity, increasing social isolation, and limiting opportunities for cognitive engagement [9, 17, 18].

Despite these insights, there remains a lack of comprehensive, China-specific data on cognitive impairment among high-risk populations. Addressing this gap is essential between understanding cognitive health challenges in these vulnerable groups and developing targeted interventions to mitigate risk and enhance cognitive resilience.

Hainan Province, China’s southernmost province, is internationally recognized as the “*World Longevity Island*” due to its high concentration of centenarians, with a prevalence of 18.75 per 100,000 people [19]. As of 2022, Hainan’ population exceeded 10 million, including approximately 1.45 million individuals aged ≥ 60 years. In response to the challenges posed by rapid population aging, the Hainan Health Commission implemented the first province-wide cognitive screening initiative, specially targeting high-risk community-dwelling older adults. This study aimed to examine cognitive status and associated risk factors in these high-risk groups to inform tailored interventions. The study design and reporting adhere to the Strengthening the Reporting of Observational Studies in Epidemiology (STROBE) guidelines [20].

## Methods

### Study design and participants

This population-based cross-sectional study was conducted in 2022 across all regions of Hainan Province, excluding Sansha (approximately 2,000 inhabitants). The study targeted community-dwelling individuals aged 60 and above who had resided in Hainan for at least six months and at high risk, i.e., with at least one of the five registry-based (Hainan Basic Public Health System, Hainan-BPHS) or self-reported health conditions: limitations in daily physical activities (physical ADLs), disability, stroke history, mental health symptoms, or subjective memory concerns. The identification of these five health conditions is described in eText 1 in Supplement 1.

The Hainan-BPHS, part of the broader Chinese BPHS program established in 2009 [21], systematically records individuals ≥ 60 years who undergo annual government-funded health checkups. Except health conditions, the registry also includes information on age, sex, home address, phone contact, etc. Using the unique civil registration number assigned to Chinese citizens at birth, individuals’ health data can be linked across regional, provincial, and national registers.

### Sampling strategy

To ensure representativeness among the high-risk older adult population, the Hainan Health Commission employed a stratified sampling method across 18 cities and counties in the province. Sample sizes for each city and county were determined proportionally based on the population aged ≥ 60 years in each region and the proportion of people with potential disability. This approach aimed at achieving a minimum sample size of 196,000 individuals. The target sample size was estimated based on a government report with estimation that approximately 19.3% of older residents in Hainan may live with functional dependence [19].

### Exposures and measures

Participants’ self-reported demographic characteristics (marital status, education, current occupation, and residence location) and other health conditions, which were assessed as potential risk factors for cognitive outcomes.

Age and sex were verified using the unique civil registration card. Age was categorized in 10-year intervals. Marital status was classified as “Married” and “Non-married” (including widowed, divorced, separated, and never married/single).

Reflecting China’s retirement norms (55 years for women and 60 years for men), participants reported any current employment status. Occupation was categorized into four groups: “White-collar” (government employees, professionals, company or organization managers, office workers), “Blue/pink-collar” (manual laborers, service industry workers), “Farmer /homemaker”, and “Retired with no other jobs”.

Education levels were categorized based on the highest years of attainment into five categories: “Illiteracy”, “Primary school (1–6 years)”, “Middle school (7–9 years)”, “High school (10–12 years)”, or “College and above (≥ 13 years)”.

Residence location was classified based on the Gross Domestic Product (GDP) tertiles of Hainan in 2023: “High GDP group” (GDP ≥ 37.97 billion yuan, including Haikou, Danzhou, Sanya, Chengmai, Qionghai, and Wenchang), “Median GDP group” (GDP 13.51–37.96 billion Yuan, including Wanning, Lingshui, Lingao, Dongfang, Ledong, Changjiang, and Anding), and “Low GDP group” (GDP < 13.51 billion yuan, including Tunchang, Baoting, Qiongzhong, Baisha, and Wuzhishan) [22].

Participants self-reported 15 physician-diagnosed health conditions, with the analysis focusing on those with a prevalence exceeding 1%: hypertension, diabetes, family dementia history, hyperlipidemia, coronary heart disease, cerebral hemorrhage, cerebral ischemia, hearing impairment, and vision impairment. Comorbidities (excluding family dementia history) were grouped by the number of these conditions: 0, 1–2, or ≥ 3.

### Cognitive outcomes and measures

Cognitive status, including cognitive normal, Pre-MCI, and MCI, were screened and assessed using the Brain Rejuvenation Initiative Study Brain Health System (BABRI-BHS), developed by the State Key Laboratory of Cognitive Neuroscience and Learning of Beijing Normal University. This system includes a range of tests assessing various cognitive function domains and has been validated for its efficacy in community elderly cognitive screening and assessment [23]. It is recommended by over 70 authoritative experts across China for large-scale screening in diverse healthcare settings including memory clinics, community health centers, and elderly care institutions [24]. The detailed description of BARBI-BHS for cognitive function screening and assessment has been previously published [23], and simply abstracted in eText 2 in Supplement 1. In brief, the system comprises three sequential sessions: SCREEN, ASSESS, and DIAGNOSE.

SCREEN: This first session collects information on older adults’ subjective cognitive complaints and general cognitive performance, taking approximately six minutes. If an older adult passes the SCREEN session, the system categorizes them as “cognitive decline unlikely” (defined as “cognitively normal” in our manuscript) and provides general health reinforcement advice. If an individual does not pass the SCREEN session, they are recommended to proceed to the ASSESS session.

ASSESS: In this session, older adults complete questionnaires covering their physical and mental health and lifestyle, and receive a more detailed cognitive evaluation using composite tests administered by trained testers. If an older adult fails the SCREEN session but passes the ASSESS session, they are categorized as “cognitive decline likely” (defined as “Pre-MCI” in our manuscript). The system then generates individualized recommendations based on the combined the result of the first two sessions.

DIAGNOSE: Older adults who do not pass the ASSESS session are advised to undergo the DIAGNOSE session, which is conducted primarily in hospitals. This session involves neuroimaging scans, blood tests, and other necessary clinical examinations to verify any pathological manifestations related to cognitive impairment. Older adults who complete the DIAGNOSE session but show no pathological findings are considered at “high risk for MCI,” meaning they exhibit significant cognitive decline but lack clear evidence of dementia. However, it is important to clarify that the DIAGNOSE session was not utilized in this community screening. In this practical community screening, individuals who fail the ASSESS session are therefore considered high-risk for MCI (defined as MCI in our manuscript).

### Data collection

Data collection was conducted from March to December 2022, using two primary methods: (1) centralized data collection, where primary healthcare workers scheduled participants for face-to-face surveys at local community or healthcare centers via telephone, and (2) household data collection, where healthcare workers made appointments by phone or direct home visits to conduct surveys. Home visits were facilitated by the familiarity of local primary care personnel with household members.

A total of 3,438 healthcare workers, comprising clinicians, nurses, primary care personnel, community workers, social workers, and volunteers, used electronic surveys on centralized tablets or mobile phones (with data costs reimbursed) to collect personal data and administer the BABRI-BHS cognitive screening and assessment.

### Bias and quality control

To ensure data reliability avoiding interviewer bias, prior to data collection, the healthcare workers in two groups underwent standardized and structured training from March 19 to March 27 and April 23 to July 10, 2022. The training covered theoretical and practical aspects, and post-training competency evaluations ensured that only qualified healthcare workers participated in data collection.

Two quality control methods were implemented during data collection:

1. Real-time data quality monitoring: A cloud-based platform supported by the provincial government provides real-time data monitoring and integrated screening endpoints accessible via mobile applications. Valid data had to fulfill several criteria: (i) successful upload of a photograph depicting the data collection scenario; (ii) survey completion in over 4 min; (iii) adherence to a pre-determined sequence of survey questions; (iv) participant receipt of the assessment report via Wechat, email, or relatives/the healthcare work’s email for print; and (v) survey conducted between 06:00–24:00, Monday to Sunday. Non-compliance resulted in a flagged entry, necessitating a re-survey and data re-entry by the healthcare worker whenever possible.
2. Field quality control: Expert teams from the Hainan Health Commission conducted on-site quality checks at community health centers and during household visits, providing immediate feedback and necessary corrections.

### Statistical analysis

Participants’ demographic characteristics were analyzed and stratified by sex and cognitive status.

For logistic regression analysis, we grouped participants aged ≥ 100 years (0.1%) into the 90 + age category when applicable, and regrouped education into four categories as illiteracy, primary school, middle school, and high school or above. Two multivariable logistic regression models were used to assess associations between participant characteristics and cognitive decline as well as MCI. Model 1 adjusted for age group, sex, marital status, education, current occupation, and residence location. Model 2 adjusted for variables in Model 1 and other chronic conditions (family dementia history, hypertension, diabetes, hyperlipidemia, chronic heart diseases, cerebral hemorrhage, ischemia, hearing impairment, vision impairment, and comorbidities). Subgroup analysis was conducted using the fully adjusted variables from Model 2 to understand sex-specific differences in observed associations.

Results were presented as Odds Ratios (ORs) with 95% confidence intervals (CIs). All statistical analyses were performed using SPSS software (version 25.0) with a two-tailed *P*-value < 0.05 considered statistically significant.

## Results

**Fig. 1:**
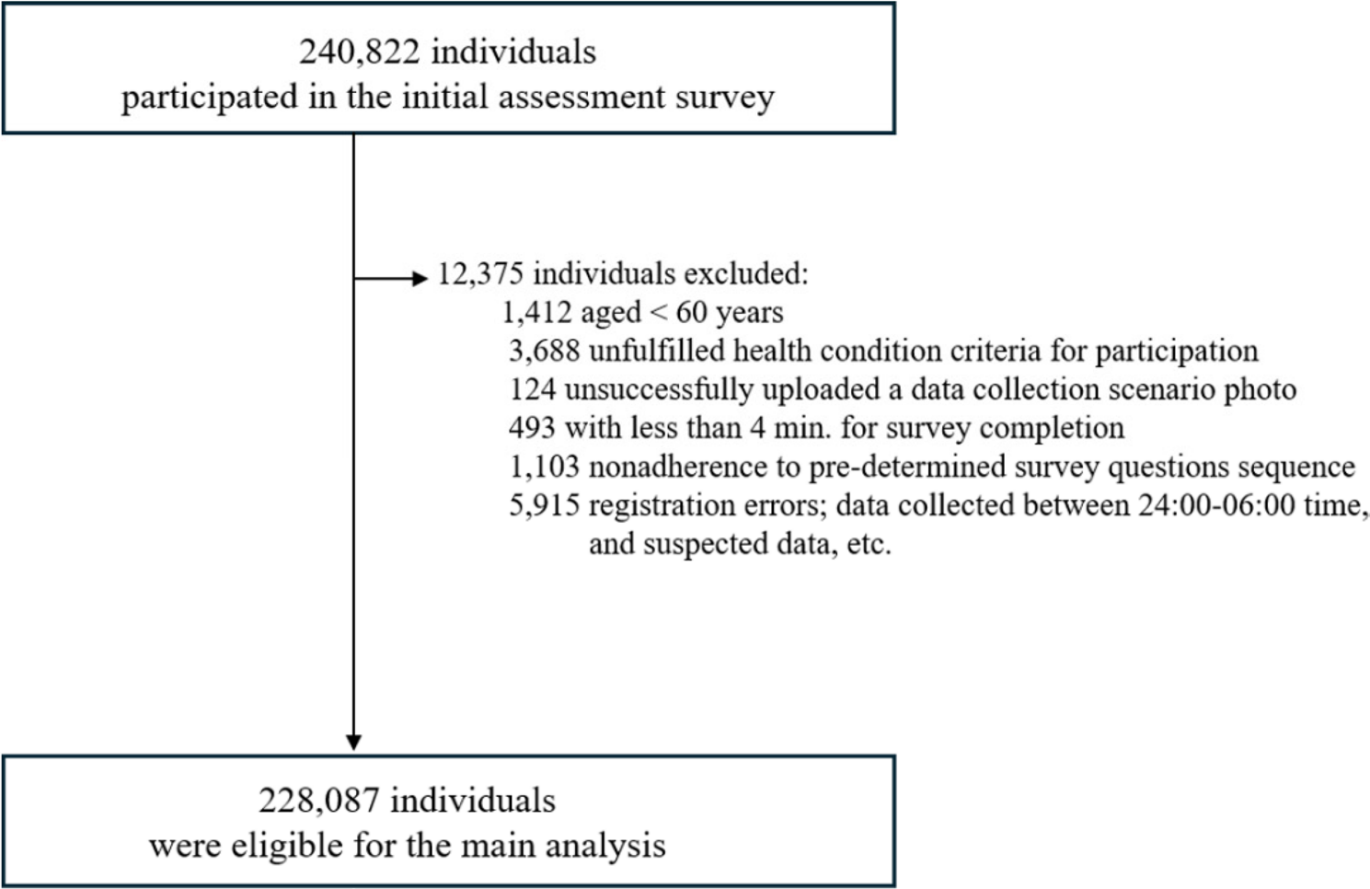
The flowchart of study participant selection

A total of 240,822 individuals participated in the initial screening and assessment. After excluding 12,357 participants due to insufficient data quality, 228,087 individuals were included in the final analysis (Fig. 1). Among these, 36,416 (16.0%) had physical ADLs, 83,478 (36.6%) were disabled, 16,630 (7.3%) had a stroke history, 13,579 (6.0%) reported mental health symptoms, and 204,871 (89.8%) reported subjective memory concerns.

The participants (mean age 73.6 ± 8.6 years) were predominantly females (57.6%), and 86.3% were married. Educationally, 28.3% had a primary education and 38.8% had no education, with females nearly twice as likely as males to be illiterate. Occupationally, more than half of the participants (57.8%) were farmers or housemakers. Hypertension was the most common physician’s diagnosed health condition, affecting 45.5% of participants, followed by vision impairment (21.7%) and hearing impairment (17.1%). Females had higher rates of both hypertension and vision impairment than males (Table 1).

**Table 1:** Sociodemographic and clinical characteristics of the study population by sex

*N* (%)	Total	Male	Female
	*n* = 228,087 (100)	*n* = 96,786 (42.4)	*n* = 131,301 (57.6)
Age in years, mean (SD)	73.6 (8.6)	73.1 (8.2)	74.1 (8.8)
Age group			
60 ~ 69	85,729 (37.6)	38,002 (39.3)	47,727 (36.3)
70 ~ 79	82,682 (36.3)	36,512 (37.7)	46,170 (35.2)
80 ~ 89	49,807 (21.8)	19,121 (19.7)	30,686 (23.4)
90 ~ 99	9,588 (4.2)	3,092 (3.2)	6,496 (4.9)
≥100	281 (0.1)	59 (0.1)	222 (0.2)
Marital status			
Married	196,942 (86.3)	88,883 (91.8)	108,059 (82.3)
Non-married status^a^	31,145 (13.7)	7,903 (8.2)	23,242 (17.7)
Educational level^b^			
Illiteracy	88,608 (38.8)	23,212 (24.0)	65,396 (49.9)
Primary school	64,543 (28.3)	26,278 (27.2)	38,265 (29.1)
Middle school	45,801 (20.1)	27,750 (28.6)	18,051 (13.7)
High school	24,438 (10.7)	16,166 (16.7)	8,272 (6.3)
College and above	4,697 (2.1)	3,380 (3.5)	1,317 (1.0)
Occupation^c^			
White-collar	17,114 (7.5)	8,646 (8.9)	8,468 (6.4)
Blue/Pink-collar	24,653 (10.8)	10,687 (11.0)	13,966 (10.6)
Farmer/homemaker	134,811 (59.1)	55,035 (56.9)	79,776 (60.8)
Retired with no current other jobs	51,509 (22.6)	22,418 (23.2)	29,091 (22.2)
City/county of residence^d^			
High GDP group	142,118 (62.3)	61,404 (63.5)	80,714 (61.5)
Medium GDP group	64,684 (28.4)	26,459 (27.3)	38,225 (29.1)
Low GDP group	21,285 (9.3)	8,923 (9.2)	12,362 (9.4)
Other health condition^e^			
Hypertension	103,727 (45.5)	43,125 (44.6)	60,602 (46.2)
Vision impairment	49,520 (21.7)	19,215 (19.9)	30,305 (23.1)
Hearing impairment	38,999 (17.1)	16,612 (17.2)	22,387 (17.1)
Diabetes mellitus	22,456 (9.8)	9,098 (9.4)	13,358 (10.2)
Hyperlipidemia	16,237 (7.1)	6,772 (7.0)	9,465 (7.2)
Coronary heart disease	11,832 (5.2)	4,988 (5.2)	6,844 (5.2)
Cerebral ischemia	7,612 (3.3)	3,328 (3.4)	4,284 (3.3)
Family dementia history	3,560 (1.6)	1,477 (1.5)	2,083 (1.6)
Cerebral hemorrhage	3,052 (1.3)	1,790 (1.8)	1,262 (1.0)
Comorbidity^f^			
0	81,124 (35.6)	35,655 (36.9)	45,649 (34.7)
1–2	120,156 (52.7)	50,144 (51.8)	70,012 (53.3)
≥3	26,696 (11.7)	10,939 (11.3)	15,757 (12.0)
Participants with^g^			
Physical ADLs	36,416 (16.0)	15,144 (15.6)	21,272 (16.2)
Disability	83,478 (36.6)	36,171 (37.4)	47,307 (36.0)
Stroke history	16,630 (7.3)	8,394 (8.7)	8,236 (6.3)
Mental health symptoms	13,579 (6.0)	5,671 (5.9)	7,908 (6.0)
Subjective memory concern	204,871 (89.8)	85,587 (88.4)	119,284 (90.8)

*Physical ADLs* limitations in activities in daily living related to physical functioning, *SD* Standard deviation

^a^Non-married status included widowed, divorced, separated, and never married/single

^b^Primary school (1–6 years), Middle school (7–9 years), High school (10–12 years), or College and above (≥ 13 years)

^c^Occupation was self-reporting any jobs currently undertaken

^d^City/county of residence were grouped based on GDP tertiles of Hainan in 2023: “High GDP group” (GDP ≥ 37.97 billion yuan, including Haikou, Danzhou, Shanya, Chengmai, Qionghai, and Wenchang), “Median GDP group” (GDP 13.51–37.96 billion Yuan, including Wanning, Lingshui, Lingao, Dongfang, Ledong, Changjiang, and Anding), and “Low GDP group” (GDP < 13.51 billion yuan, including Tunchang, Baoting, Qiongzhong, Baisha, and Wuzhishan)

^e^The percentage of a single disease among the population

^f^Comorbidity was categorized according to the number of “Other health conditions” (except family dementia history) in any combination

^g^The percentage of individuals with each single disease among the population

Table 2 shows that among the 228,087 participants, 117,304 (51.4%) were assessed as having Pre-MCI, and 72,917 (32.0%) as having MCI. Overall, the prevalence of Pre-MCI and MCI was higher among individuals who were older, had lower education attainment, were farmers by occupation, or had chronic conditions, than those with normal cognition. Additionally, individuals with a family history of dementia, a history of cerebral hemorrhage, or physical ADL had a higher prevalence of MCI than Pre-MCI or normal cognition.

**Table 2:** Sociodemographic and clinical characteristics of the study population by cognitive status

N (100%)	Cognitive Status			
Total	Normal	Pre-MCI	MCI	p-value
*n* = 228,087 (100)	*n* = 37,866 (16.6)	*n* = 117,304 (51.4)	*n* = 72,917 (32.0)	
Age, mean (SD)	70.2 (7.0)	73.7 (8.4)	75.4 (9.1)	< 0.001
Age group				< 0.001
60–69	19,848 (23.2)	42,108 (49.1)	23,773 (27.7)	
70–79	13,698 (16.5)	45,203 (54.7)	23,781 (28.8)	
80–89	3,946 (7.9)	25,529 (51.3)	20,332 (40.8)	
90–99	371 (3.9)	4,338 (45.2)	4,879 (50.9)	
≥100	3 (1.1)	126 (44.8)	152 (54.1)	
Sex				< 0.001
Male	18,656 (19.3)	50,210 (51.9)	27,920 (28.8)	
Female	19,210 (14.6)	67,094 (51.1)	44,997 (34.3)	
Marital status				< 0.001
Married	34,969 (17.8)	102,753 (52.2)	59,220 (30.0)	
Non-married status^a^	2,897 (9.3)	14,551 (46.7)	13,697 (44.0)	
Educational level^b^				< 0.001
No education	6,441 (7.3)	45,536 (51.4)	36,631 (41.3)	
Primary school	9,639 (14.9)	34,625 (53.6)	20,279 (31.5)	
Middle school	11,581 (25.3)	23,454 (51.2)	10,766 (23.5)	
High school	8,224 (33.6)	11,649 (47.7)	4,565 (18.7)	
College and above	1,981 (42.2)	2,040 (43.4)	676 (14.4)	
Occupation^c^				< 0.001
White collar	3,812 (22.3)	8,936 (52.2)	4,366 (25.5)	
Retired with no other jobs	11,768 (22.8)	25,379 (49.3)	14,362 (27.9)	
Blue/Pink-collar	5,186 (21.0)	11,876 (48.2)	7,591 (30.8)	
Farmer/housemaker	17,100 (12.7)	71,113 (52.8)	46,598 (34.5)	
City/county of residence^d^				< 0.001
High GDP cities	26,872 (18.9)	74,694 (52.6)	40,552 (28.5)	
Medium GDP cities	7,049 (10.9)	33,338 (51.5)	24,297 (37.6)	
Low GDP cities	3,945 (18.5)	9,272 (43.6)	8,068 (37.9)	
Other chronic condition				
Hypertension				< 0.001
No	22,334 (18.0)	64,971 (52.2)	37,055 (29.8)	
Yes	15,532 (15.0)	52,333 (50.4)	35,862 (34.6)	
Vision impairment				< 0.001
No	32,170 (18.0)	94,054 (52.7)	52,329 (29.3)	
Yes	5,694 (11.5)	23,243 (46.9)	20,583 (41.6)	
Hearing impairment				< 0.001
No	34,677 (18.4)	99,321 (52.5)	55,078 (29.1)	
Yes	3,188 (8.2)	17,976 (46.1)	17,835 (45.7)	
Diabetes mellitus				< 0.001
No	33,824 (16.5)	106,601 (51.8)	65,192 (31.7)	
Yes	4,040 (18.0)	10,695 (47.6)	7,721 (34.4)	
Coronary heart disease				< 0.001
No	35,971 (16.6)	111,891 (51.8)	68,375 (31.6)	
Yes	1,894 (16.0)	5,400 (45.6)	4,538 (38.4)	
Hyperlipidemia				< 0.001
No	34,820 (16.4)	109,495 (51.7)	67,519 (31.9)	
Yes	3,046 (18.8)	7,800 (48.0)	5,391 (33.2)	
Cerebral ischemia				< 0.001
No	36,807 (16.7)	113,896 (51.7)	69,751 (31.6)	
Yes	1,056 (13.9)	3,395 (44.6)	3,161 (41.5)	
Family dementia history				< 0.001
No	37,478 (16.7)	115,821 (51.6)	71,228 (31.7)	
Yes	388 (10.9)	1,483 (41.7)	1,689 (47.4)	
Cerebral hemorrhage				< 0.001
No	37,597 (16.7)	116,066 (51.6)	71,356 (31.7)	
Yes	267 (8.7)	1,229 (40.3)	1,556 (51.0)	
Comorbidity^e^				< 0.001
0	15,825 (19.5)	44,126 (54.4)	21,173 (26.1)	
1–2	19,193 (16.0)	61,172 (50.9)	39,791 (33.1)	
≥ 3	2,837 (10.6)	11,940 (44.7)	11,919 (44.7)	
Types of participants				
Physical ADLs	2,208 (6.1)	16,607 (45.6)	17,601 (48.3)	< 0.001
Disabled	9,100 (10.9)	42,099 (50.4)	32,279 (38.7)	< 0.001
Stroke history	1,957 (11.8)	7,456 (44.8)	7,217 (43.4)	< 0.001
Mental health symptoms	816 (6.0)	6,689 (49.3)	6,074 (44.7)	< 0.001
Subjective memory concerns	33,916 (16.5)	103,435 (50.5)	67,520 (33.0)	< 0.001

*Physical ADLs* limitations in activities in daily living related to physical functioning, *MCI* Mild cognitive impairment, *SD* Standard deviation

^a^Non-married status included widowed, divorced, separated, and never married/single

^b^Primary school (1–6 years), Middle school (7–9 years), High school (10–12 years), or College and above (≥ 13 years)

^c^Occupation was self-reporting any jobs currently undertaken

^d^City/county of residence were grouped based on GDP tertiles of Hainan in 2023: “High GDP group” (GDP ≥ 37.97 billion yuan, including Haikou, Danzhou, Shanya, Chengmai, Qionghai, and Wenchang), “Median GDP group” (GDP 13.51 − 37.96 billion Yuan, including Wanning, Lingshui, Lingao, Dongfang, Ledong, Changjiang, and Anding), and “Low GDP group” (GDP < 13.51 billion yuan, including Tunchang, Baoting, Qiongzhong, Baisha, and Wuzhishan)

^e^Comorbidity was categorized according to the number of “Other health conditions” (except family dementia history) in any combination

**Fig. 2:**
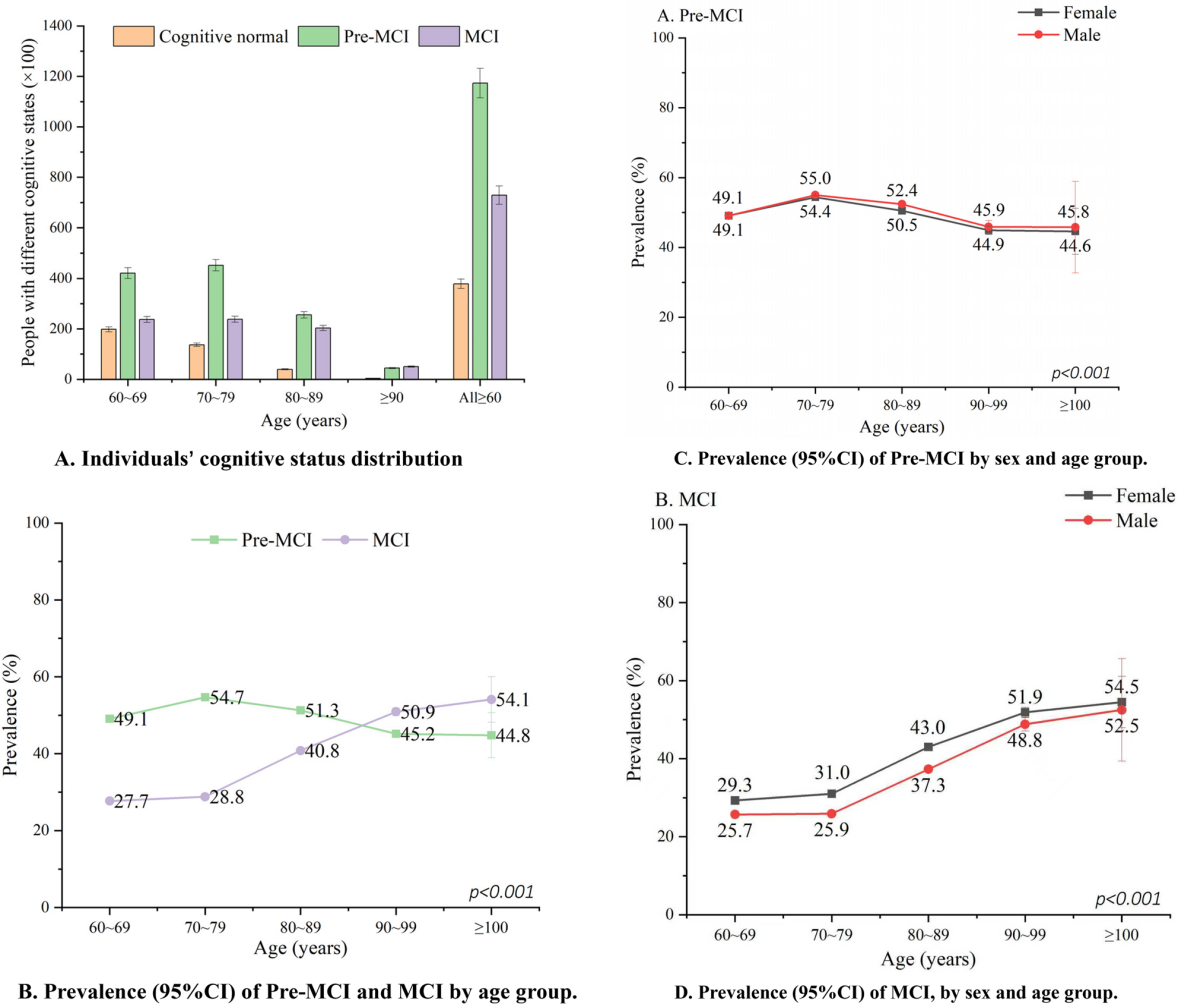
Cognitive status distribution among study population

Cognitive status assessment showed a predominance of Pre-MCI and MCI across all age groups. Age group analysis revealed an ascending prevalence trend in Pre-MCI from the age group 60–69 to 70–79, followed by a decreasing trend. Conversely, MCI consistently increased from ages 70–79, becoming notably prevalent in older age groups (Fig. 2A and D).

Using logistic regression analysis adjusted for a comprehensive set of covariates (Model 2), several demographic and health-related factors showed significant associations with cognitive outcomes. Advancing age was strongly linked to increased odds of both Pre-MCI (ORs ranged from 1.27 [95% CI: 1.24–1.30] to 3.09 [2.77–3.45]) and MCI (ORs ranged from 1.04 [1.01–1.07] to 4.28 [3.84–4.78]). Lower education levels were also associated with higher risks, with ORs ranging from 1.37 [1.32–1.41] to 3.74 [3.59–3.89] for Pre-MCI, and from 1.61 [1.54–1.68] to 6.92 [6.60–7.25] for MCI. Non-married status (OR 1.07 [1.03–1.12] for Pre-MCI; 1.45 [1.39–1.52] for MCI), residence in medium GDP regions (OR 1.74 [1.66–1.82] for Pre-MCI; 1.38 [1.32–1.45] for MCI), and occupation as farmer/housemaker (OR 1.37 [1.31–1.43] for Pre-MCI; 1.59 [1.51–1.67] for MCI) were also significantly associated with increased risk. Regarding health conditions, cerebral hemorrhage (OR 1.56 [1.36–1.80] for Pre-MCI; 2.97 [2.58–3.41] for MCI), hearing impairment (OR 1.47 [1.39–1.55] for Pre-MCI; 2.01 [1.90–2.13] for MCI), family dementia history (OR 1.15 [1.03–1.29] for Pre-MCI; 1.84 [1.63–2.07] for MCI), and hypertension (OR 1.15 [1.10–1.20] for Pre-MCI; 1.22 [1.16–1.29] for MCI) showed strong associations with both Pre-MCI and MCI. Other conditions, including diabetes (OR 1.11 [1.05–1.17]), coronary heart disease (OR 1.09 [1.02–1.16]), cerebral ischemia (OR 1.33 [1.22–1.44]), and vision impairment (OR 1.19 [1.13–1.25]) were significantly associated with MCI only. These findings underscore the multifactorial nature of cognitive impairment risk among older adults (Table 3).

**Table 3:** Logistic regression of covariates with cognitive outcomes in older adults aged 60 and above

	Cognitive status OR (95% CIs)			
	Model 1		Model 2	
	Pre-MCI	MCI	Pre-MCI	MCI
Age group				
60–69	1 (ref)	1 (ref)	1 (ref)	1 (ref)
70–79	1.30 (1.26–1.33)	1.11 (1.08–1.14)	1.27 (1.24–1.30)	1.04 (1.01–1.07)
80–89	2.26 (2.17–2.35)	2.69 (2.59–2.81)	2.13 (2.05–2.21)	2.31 (2.22–2.41)
≥ 90	3.39 (3.04–3.77)	5.36 (4.80–5.98)	3.09 (2.77–3.45)	4.28 (3.84–4.78)
Sex				
Female	1 (ref)	1 (ref)	1 (ref)	1 (ref)
Male	1.06 (1.04–1.09)	1.06 (1.03–1.09)	1.05 (1.02–1.08)	1.05 (1.02–1.08)
Marital status				
Married	1 (ref)	1 (ref)	1 (ref)	1 (ref)
Non-married status^a^	1.08 (1.04–1.13)	1.48 (1.41–1.54)	1.07 (1.03–1.12)	1.45 (1.39–1.52)
Educational level^b^				
High school or above	1 (ref)	1 (ref)	1 (ref)	1 (ref)
Middle school	1.37 (1.32–1.42)	1.59 (1.52–1.66)	1.37 (1.32–1.41)	1.61 (1.54–1.68)
Primary school	2.13 (2.05–2.21)	2.97 (2.84–3.10)	2.12 (2.04–2.20)	3.02 (2.89–3.16)
Illiteracy (no education)	3.76 (3.61–3.92)	6.79 (6.48–7.11)	3.74 (3.59–3.89)	6.92 (6.60–7.25)
Occupation^c^				
White collar	1 (ref)	1 (ref)	1 (ref)	1 (ref)
Retired with no other jobs	0.90 (0.86–0.95)	1.04 (0.98–1.09)	0.90 (0.86–0.94)	1.05 (0.99–1.11)
Blue/Pink-collar	1.04 (0.98–1.09)	1.32 (1.25–1.41)	1.03 (0.97–1.08)	1.28 (1.21–1.36)
Farmer/housemaker	1.38 (1.32–1.44)	1.58 (1.50–1.66)	1.37 (1.31–1.43)	1.59 (1.51–1.67)
Residence location^d^				
High GDP group	1 (ref)	1 (ref)	1 (ref)	1 (ref)
Medium GDP group	1.76 (1.68–1.84)	1.41 (1.34–1.48)	1.74 (1.66–1.82)	1.38 (1.32–1.45)
Low GDP group	1.34 (1.29–1.40)	0.87 (0.83–0.91)	1.36 (1.30–1.41)	0.90 (0.86–0.94)
Other chronic condition				
Cerebral hemorrhage			1.56 (1.36–1.80)	2.97 (2.58–3.41)
Hearing impairment			1.47 (1.39–1.55)	2.01 (1.90–2.13)
Family dementia history			1.15 (1.03–1.29)	1.84 (1.63–2.07)
Hypertension			1.15 (1.10–1.20)	1.22 (1.16–1.29)
Cerebral ischemia			1.02 (0.94–1.10)	1.33 (1.22–1.44)
Vision impairment			1.02 (0.98–1.07)	1.19 (1.13–1.25)
Diabetes mellitus			0.97 (0.92–1.02)	1.11 (1.05–1.17)
Hyperlipidemia			0.97 (0.92–1.03)	1.03 (0.97–1.09)
Coronary heart disease			0.90 (0.85–0.96)	1.09 (1.02–1.16)
Comorbidity^e^				
0			1 (ref)	1 (ref)
1–2			0.97 (0.92–1.03)	1.07 (1.01–1.13)
≥ 3			1.00 (0.89–1.13)	1.11 (0.98–1.26)

*MCI* Mild cognitive impairment, *OR* Odds ratio, *95% CI* 95% confidence interval, *Physical ADLs* limitations in activities in daily living related to physical functioning, *SD* Standard deviation

^a^Non-married status included widowed, divorced, separated, and never married/single

^b^Primary school (1–6 years), Middle school (7–9 years), High school and above (≥ 10 years)

^c^Occupation was self-reporting any jobs currently undertaken

^d^City/county of residence were grouped based on GDP tertiles of Hainan in 2023: “High GDP group” (GDP ≥ 37.97 billion yuan, including Haikou, Danzhou, Shanya, Chengmai, Qionghai, and Wenchang), “Median GDP group” (GDP 13.51–37.96 billion Yuan, including Wanning, Lingshui, Lingao, Dongfang, Ledong, Changjiang, and Anding), and “Low GDP group” (GDP < 13.51 billion yuan, including Tunchang, Baoting, Qiongzhong, Baisha, and Wuzhishan)

^e^Comorbidity was categorized according to the number of “Other health condition” (except family dementia history) (0, 1–2, or ≥ 3) in any combination

**Fig. 3:**
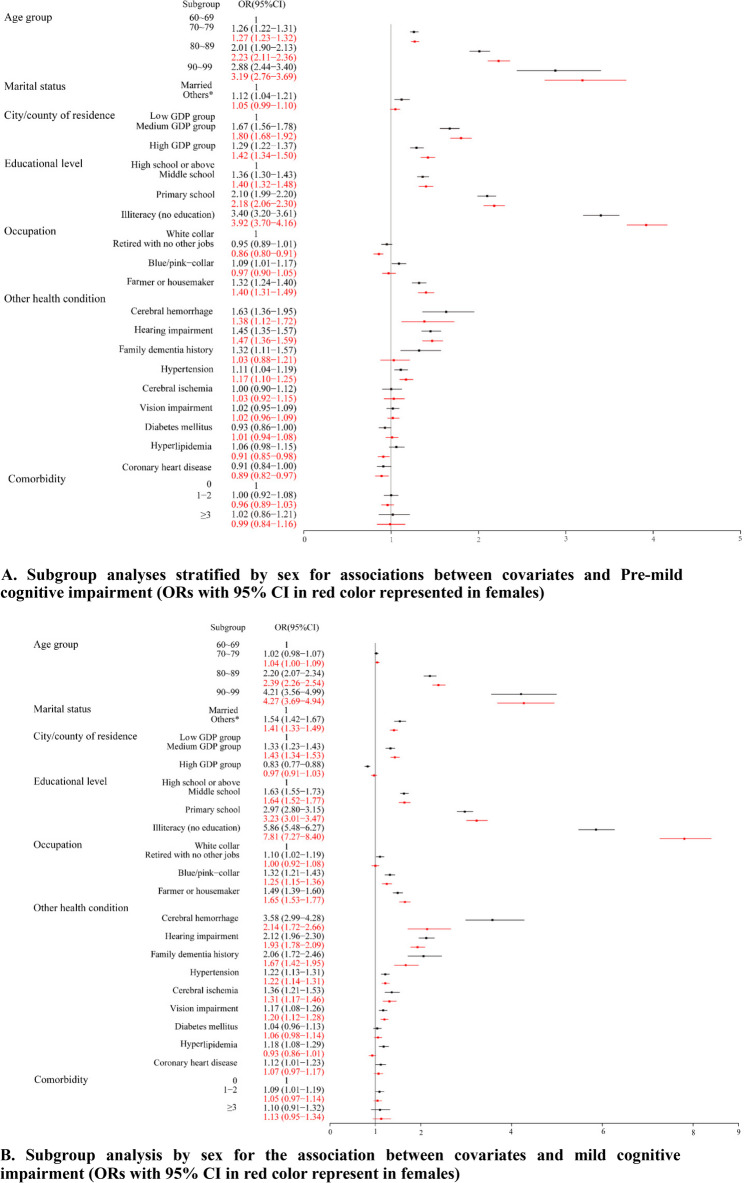
Subgroup analyses stratified by sex for associations between covariates and Pre-mild cognitive impairment (Pre-MCI) and MCI. **A**. Associations between covariates and Pre-MCI by sex. **B**. Associations between covariates and MCI by sex

Sex-stratification analysis showed that associations between various factors and cognitive status were more pronounced in females for age, education, and residence location, while marital status, cerebral hemorrhage, hearing impairment, and family dementia history showed stronger associations in males (Fig. 3A and B).

Comorbidity was not identified as a risk factor in either the overall analysis showed in Table 3 or the sex-stratified subgroup analyses in the Table 4.

**Table 4:** Subgroup analyses stratified by sex for associations between covariates and cognitive status in older adults aged 60 and above in Hainan Province, the tropical region of China

	OR (95% CI)			
	Pre-MCI		MCI	
	Males	Females	Males	Females
Age group				
60–69	1 (ref)	1 (ref)	1 (ref)	1 (ref)
70–79	1.26 (1.22–1.31)	1.27 (1.23–1.32)	1.02 (0.98–1.07)	1.04 (1.00-1.09)
80–89	2.01 (1.90–2.13)	2.23 (2.11–2.36)	2.20 (2.07–2.34)	2.39 (2.26–2.54)
≥ 90	2.88 (2.44–3.40)	3.19 (2.76–3.69)	4.21 (3.56–4.99)	4.27 (3.69–4.94)
Marital status^a^				
Married	1 (ref)	1 (ref)	1 (ref)	1 (ref)
Non-married status	1.12 (1.04–1.21)	1.05 (0.99–1.10)	1.54 (1.42–1.67)	1.41 (1.33–1.49)
Educational level^b^				
High school or above	1 (ref)	1 (ref)	1 (ref)	1 (ref)
Middle school	1.36 (1.30–1.43)	1.40 (1.32–1.48)	1.63 (1.55–1.73)	1.64 (1.52–1.77)
Primary school	2.10 (1.99–2.20)	2.18 (2.06–2.30)	2.97 (2.80–3.15)	3.23 (3.01–3.47)
Illiteracy (no education)	3.40 (3.20–3.61)	3.92 (3.70–4.16)	5.86 (5.48–6.27)	7.81 (7.27–8.40)
Occupation^c^				
White collar	1 (ref)	1 (ref)	1 (ref)	1 (ref)
Retired with no other jobs	0.95 (0.89–1.01)	0.86 (0.80–0.91)	1.10 (1.02–1.19)	1.00 (0.92–1.08)
Blue/pink-collar	1.09 (1.01–1.17)	0.97 (0.90–1.05)	1.32 (1.21–1.43)	1.25 (1.15–1.36)
Farmer or housemaker	1.32 (1.24–1.40)	1.40 (1.31–1.49)	1.49 (1.39–1.60)	1.65 (1.53–1.77)
City/county of residence^d^				
Low GDP group	1 (ref)	1 (ref)	1 (ref)	1 (ref)
Medium GDP group	1.67 (1.56–1.78)	1.80 (1.68–1.92)	1.33 (1.23–1.43)	1.43 (1.34–1.53)
High GDP group	1.29 (1.22–1.37)	1.42 (1.34–1.50)	0.83 (0.77–0.88)	0.97 (0.91–1.03)
Other health condition				
Cerebral hemorrhage	1.63 (1.36–1.95)	1.38 (1.12–1.72)	3.58 (2.99–4.28)	2.14 (1.72–2.66)
Hearing impairment	1.45 (1.35–1.57)	1.47 (1.36–1.59)	2.12 (1.96–2.30)	1.93 (1.78–2.09)
Family dementia history	1.32 (1.11–1.57)	1.03 (0.88–1.21)	2.06 (1.72–2.46)	1.67 (1.42–1.95)
Hypertension	1.11 (1.04–1.19)	1.17 (1.10–1.25)	1.22 (1.13–1.31)	1.22 (1.14–1.31)
Cerebral ischemia	1.00 (0.90–1.12)	1.03 (0.92–1.15)	1.36 (1.21–1.53)	1.31 (1.17–1.46)
Vision impairment	1.02 (0.95–1.09)	1.02 (0.96–1.09)	1.17 (1.08–1.26)	1.20 (1.12–1.28)
Diabetes mellitus	0.93 (0.86-1.00)	1.01 (0.94–1.08)	1.04 (0.96–1.13)	1.06 (0.98–1.14)
Hyperlipidemia	1.06 (0.98–1.15)	0.91 (0.85–0.98)	1.18 (1.08–1.29)	0.93 (0.86–1.01)
Coronary heart disease	0.91 (0.84-1.00)	0.89 (0.82–0.97)	1.12 (1.01–1.23)	1.07 (0.97–1.17)
Comorbidity^e^				
0	1 (ref)	1 (ref)	1 (ref)	1 (ref)
1–2	1.00 (0.92–1.08)	0.96 (0.89–1.03)	1.09 (1.01–1.19)	1.05 (0.97–1.14)
≥ 3	1.02 (0.86–1.21)	0.99 (0.84–1.16)	1.10 (0.91–1.32)	1.13 (0.95–1.34)

*MCI *Mild cognitive impairment, *OR* Odds ratio, *95% CI* 95% confidence interval, *Physical ADLs* limitations in activities in daily living related to physical functioning, *SD* Standard deviation

^a^Non-married status included widowed, divorced, separated, and never married/single

^b^Primary school (1–6 years), Middle school (7–9 years), High school and above (≥ 10 years)

^c^Occupation was self-reporting any jobs currently undertaken

^d^City/county of residence were grouped based on GDP tertiles of Hainan in 2023: “High GDP group” (GDP ≥ 37.97 billion yuan, including Haikou, Danzhou, Shanya, Chengmai, Qionghai, and Wenchang), “Median GDP group” (GDP 13.51–37.96 billion Yuan, including Wanning, Lingshui, Lingao, Dongfang, Ledong, Changjiang, and Anding), and “Low GDP group” (GDP < 13.51 billion yuan, including Tunchang, Baoting, Qiongzhong, Baisha, and Wuzhishan)

^e^Comorbidity was categorized according to the number of “Other health condition” except family dementia history) (0, 1–2, or ≥ 3) in any combination

## Discussion

This study provides the first large-scale, comprehensive population-based epidemiological assessment of cognitive status among high-risk older adults in China. Among 228,087 eligible participants, the prevalence of Pre-MCI and MCI was 51.4% and 32.0%, respectively.

Several demographic characteristics were significantly associated with an increased risk of Pre-MCI and MCI, including age, sex, marital status, education, geographic location, and occupation. Additionally, self-reported physician-diagnosed health conditions, particularly cerebral hemorrhage, hearing impairment, family dementia history, and hypertension, were primary contributors to both Pre-MCI and MCI. Other conditions, including diabetes, coronary heart disease, cerebral ischemia, and vision impairment, specifically increased the odds for MCI only.

Sex-specific differences were evident in the association between risk factors and cognitive outcomes. Age, education, and geographic location showed stronger associations with Pre-MCI and MCI in females, whereas marital status, cerebral hemorrhage, hearing impairment, and family dementia history were more strongly associated with cognitive outcomes in males.

Our findings indicate an MCI prevalence of 32.0%, nearly twice the prevalence reported in a 2020 study based on the 46,011 Chinese adults ≥ 60 years recruited between 2015 and 2018^2^. Despite differences in assessment methods including diagnostic criteria in MCI, this discrepancy may reflect the targeted nature of our study population, which consisted of high-risk individuals with specific health conditions. This aligns with previous research linking certain health conditions to an increased risk of MCI [25–27]. Additionally, lower educational attainment and occupational status (38.8% had only primary education, 28.3% had no education, and 57.8% were farmers or housemakers) may contribute to the high prevalence in our targets. Previous studies have linked education and cognitively demanding occupations to higher cognitive abilities, suggesting education’s key role in cognitive status [28, 29]. The high prevalence (45.5%) of hypertension, although lower than the global prevalence, may also contribute significantly [30, 31].

Demographic factors, including age, sex, education, and marital status, were significant independent risk factors for Pre-MCI and MCI, consistent with general older adults [2, 32]. Interestingly, residing in medium GDP regions was associated with higher odds for Pre-MCI and MCI, contradicting the assumption that better economic status correlates with improved cognitive function [33]. This might be explained by socioeconomic transitions, such as expanding urbanization and advancement in agriculture, prompting more farmers to relocate to urban areas in search of better employment opportunities, thereby impacting cognitive demographics. The lower odds of MCI observed in the low-GDP group may be due to underreporting or under-detection of cognitive impairment, which is more likely in areas with limited healthcare access and lower health awareness. In contrast, higher-GDP regions may have higher detection rates due to greater health literacy and better recognition and reporting of subtle symptoms. Our earlier research using population data from Denmark found similar patterns: individuals with higher income tended to receive earlier diagnoses, while those with lower socioeconomic status were more likely to receive a late diagnosis [34]. These findings underscore the complex interplay between socioeconomic factors and cognitive health, and highlight the need for context-specific strategies to improve early detection and intervention efforts.

Our findings also revealed significant contributors like cerebral hemorrhage, hearing impairment, and family dementia history, hypertension, consistent with previous studies [2, 35–37]. Some conditions, like diabetes, showed higher odds of MCI only, possibly reflecting disease progression. Contrary to expectation, varying levels of comorbidity indices did not emerge as significant risk factors for cognitive outcomes in our study population, which has typically reported positive associations previously in general older adults [38]. A prospective cohort study employed different comorbidity burden indices and found that the Charlson Comorbidity Index and Medication Regimen Complexity Index score were not linked with cognitive function, whereas the severity index of Cumulative Illness Rating Scale for Geriatrics was associated [39]. As our study is a cross-sectional study with only one-time measurements of comorbidity and cognitive function, further longitudinal studies are warranted to explore these discrepancies.

### Study limitations and implications

Several limitations of this study should be acknowledged. First, the definition of high-risk older adults was based on the presence of the selected health conditions, including physical limitations, disability, stroke history, mental health symptoms, and subjective memory concerns. While this approach allowed for efficient community-based screening, it does not capture the severity or cumulative burden of risk factors. As noted by Stephan et al. single-domain or overly simplified models often lack predictive accuracy [40]. Similarly, Barnes et al. [41]. emphasize that comprehensive risk indices incorporating multiple domains, such as cognitive, medical, genetic, and neuroimaging data, are essential for accurate risk stratification. Second, although the BABRI-BHS has been validated for cognitive assessment, it may not fully capture all aspects of cognitive function. In particular, individuals with dementia or severe cognitive impairment are often unable to complete the survey and are therefore consequently excluded, not only from high-quality research but also from essential health services [42]. Specific local dialects and participants’ particular health conditions might limit test accuracy, despite expert modifications. However, for a large-scale population-based community survey, this assessment tool is effective for comprehensive target capture within a short time duration with limited workforce resources. Furthermore, our study may overrepresent subjective memory concerns, as motivated individuals were more likely to participate. Notably, 89.8% of participants reported subjective memory concerns, higher than typical rates. Severe health conditions such as stroke, mental health symptoms, and disability might be underreported due to the survey requiring normal communication and executive capabilities. 

Lifestyle factors, including smoking, alcohol consumption, and physical activities, are important risk factors but are reversible for MCI [10]. For the consideration of survey time constraints among olde adults on a population-based large scale, the health conditions included in the analysis were based on self-reported physician diagnoses, which may be subject to recall or reporting bias. Future studies should consider validating self-reported data with medical records or clinical assessments to improve accuracy. Again, due to time constraints inherent to large-scale population-based cognitive screening, certain lifestyle factors, such as smoking, alcohol consumption, and physical activity, were not included in the present analysis, but are planned for assessment in future follow-up studies.

Moreover, the findings of this study are primarily generalizable to regions with similar demographic and administrative structures. However, the cross-sectional design limits our ability to draw causal inferences; thus, longitudinal cohort studies are needed to further explore the temporal relationships between risk factors and cognitive outcomes. Although the use of stratified sampling helped reduce regional sampling bias within the high-risk group, our inclusion criteria, which was based on selected health conditions, introduced inherent selection bias. As a result, the findings may not represent the general older adult population, but rather reflect the cognitive status and associated risk profile of a particularly vulnerable subgroup.

## Conclusion

Our study advances understanding of the interplay of demographic factors, health conditions, and sex in shaping cognitive function among older adults with specific health conditions in the sole tropical region of China. Given the high prevalence of Pre-MCI and MCI and the identified risk factors, tailored prevention and intervention strategies are essential.

## Supplementary Information

Supplementary Material 1.

## Data Availability

Data cannot be shared openly but are available on request. Access will be granted following a review of proposals and is contingent upon securing a data use agreement. The data will be accessible until December 31, 2028, a period extending to five years post-publication of this study. For inquiries or requests pertaining to the study data, please contact the corresponding author.

## Abbreviations

BABRI: Beijing Aging Brain Rejuvenation Initiative
BABRI-BHS: Beijing Aging Brain Rejuvenation Initiative Study-Brain Health System
CI: Confidence Intervals
GDP: Gross Domestic Product
Hainan-BPHS: Hainan Basic Public Health System
MCI: Mild Cognitive Impairment
OR: Odds Ratio
Physical ADL: Limitations in Daily Physical Activities

## Declarations

### Content for publication

All participants provided informed consent for the use of their personal data in research, including dissemination through publications.
