# Supplementary material for: Cognitive status and associated risk factors in high-risk older adults of China: a population-based cross-sectional study
Source: BMC Geriatr. 2026 Apr 1;26:684. doi: 10.1186/s12877-026-07412-y (PMC13185332; doi:10.1186/s12877-026-07412-y)
Supplement: Supplementary file 1 — Supplementary Material 1. [file 12877_2026_7412_MOESM1_ESM.docx]

**eText 1: Definition of physical ADLs, being disabled, mental health symptoms, and subjective memory concern**

Physical ADLs, grounded in the principles of human functional development,^1^ encompass six assessment categories: bathing, dressing, toileting, transferring, bowel and bladder control, and feeding. In this study, an individual was classified as having physical ADLs if self-reported, or reported by his/her family member, unable to perform any of these six essential tasks. If a participant was severely dependent and unable to complete the screening process, they were excluded at the discretion of the screener.

Disabled older individuals were defined as individuals registered in Hainan Basic Public Health Services (Hainan-BPHS) as disabled in vision, hearing, speech, and body parts. To capture those individuals who were disabled but without registration in Hainan-BPHS, healthcare workers have doubled to check disabled certificates through the Department of Social Welfare of Hainan Registration. In addition, during the data collection, healthcare workers also identified individuals with visual, hearing, or speech impairment, regardless of official certificate, as disabled. In cases where severe disabilities—such as significant vision or hearing impairments—interfered with the screening process, participants were excluded at the screener’s discretion.

Mental health symptoms were defined based on mild behavioral impairment that occurs in non-dementia elderly populations.^2^ In this study, individuals included for screening were those registered in the Hainan-BPHS with anxiety or depression, as well as those reported by family doctors or family members to have abnormal perception or thought content. If these symptoms were severe enough to prevent completion of the screening process, the individuals were excluded at the screener’s discretion.

Subject memory concern was referred to as individuals self-reporting or family member/s reporting on memory-related issues. This included frequently misplacing items, repetitive speech, and asking the same questions multiple times.

**Reference**

1. Katz S, Akpom CA. 12. Index of ADL. *Med Care*. 1976; 14(5 Suppl): 116-8.
2. Ruthirakuhan M, Ismail Z, Herrmann N, Gallagher D, Lanctôt KL. Mild behavioral impairment is associated with progression to Alzheimer's disease: A clinicopathological study. *Alzheimers Dement.* 2022; 18(11): 2199-208.

**eText 2: Description of the Brain Rejuvenation Initiative Study Brain Health System (BABRI-BHS) to screen and assess individuals’ cognitive function.**

The BARBI-BHS has three designed sessions: SCREEN, ASSESS, and DIAGNOSE. The SCREEN session consists of three items: the subjective cognitive evaluation (SCE), the mini -MMSE (MMSE stands for Mini Mental State Examination), and the episodic memory test (EMT).

The SCE was designed as a questionnaire with seven questions targeting five cognitive domains and mental status changes in recent years. From the original MMSE, each of the 19 items was tested in a training set to distinguish mild cognitive impairment (MCI) from normal controls. Two items—“recall of three objects” and “attention and calculation (count backward from 100 by 7s)”—had the highest AUC values, so they were selected to form the mini-MMSE, together with temporal and spatial orientation items. By shortening the total assessment time to two minutes, this new scale mitigates the MMSE’s heavy reliance on language-based tasks and reduces its dependence on educational background.

The EMT was built on the classical cognitive recognizance paradigm. Individuals were required to complete the key judgment of whether a series of images presented at a certain time were “natural objects” or “artificial objects”, and were asked to memorize these pictures, which was the coding stage. Then, some of the pictures and the newly added pictures were judged by pressing the button “appear” or “not appear”, i.e., the recognition stage. The accuracy of the EMT and the response time were recorded.

The mini-MMSE is scored as follows: if the total for Questions 1–3 is 5, Question 4 is 4 or higher, and Question 5 is 2 or higher, overall cognitive function is considered normal. Conversely, if the total for Questions 1–3 is less than 5, or Question 4 is less than 4, or Question 5 is less than 2, overall cognitive ability is deemed impaired.

For the episodic memory test, scores differ by age group. Among individuals under 60 years of age, a score below 74.3 indicates poor episodic memory, whereas a score of 74.3 or above indicates good episodic memory. For those aged 60 to 69, a score below 73.5 points to poor episodic memory, while a score of 73.5 or above suggests good episodic memory. Finally, for individuals 70 and older, a score below 71.5 indicates poor episodic memory, whereas a score of 71.5 or above signals good episodic memory.

In terms of subjective cognitive evaluation, a score under 6 signifies the presence of self-perceived cognitive decline, whereas a score of 6 or higher suggests the absence of such decline.

The most sensitive neuropsychological test to distinguish MCI from cognitively healthy adults was identified to compose the ASSESS session including the Auditory Verbal Learning test, the Trail-Making Test part A, the Rey-Osterrieth Complex Figure Test delay recall, and the Category

Verbal Fluency Test, together with questionnaires investigating the physical/mental conditions and lifestyle information.

When distinguishing cognitively impaired older adults (MCI, and dementia defined with MMSE score below 24) from cognitively healthy older adults, the canonical variable extracted from tests in the SCREEN session achieved an area under the curve (AUC) of 0·730, with a sensitivity of 0·630 and a specificity of 0·780; in the ASSESS session, the AUC was 0·906, the sensitivity was 0·809, and the specificity was 0·854.^1^

ASSESS session from the complete BABAR-BHS, which automatically pushed one of the cognitive tests composed of the ASSESS section to the individuals for further assessment.

The DIAGNOSE session was designed for clinical diagnosis and was thus not adapted for the present study.

In fact, due to well-known commercial considerations, the BABRI Brain Health System (BABRI-BHS) algorithm is not fully disclosed. This is a common issue among today’s popular AI tools, many of which aren’t entirely open source. Consequently, we can only provide the normal reference values for each specific assessment. As for how MCI is determined, our understanding is that BABRI-BHS is based on algorithms developed from extensive research and validation in the Chinese population using data from the BABRI cohort. It offers excellent convenience for community-based applications and produces screening results with strong reference value.

**Reference**

1. Yang Y, Lv C, Li H, Chen K, Li X, Chen Y, et al. Community-based Model for Dementia Risk Screening: The Beijing Aging Brain Rejuvenation Initiative (BABRI) Brain Health System. *J Am Med Dir Assoc.* 2021; 22(7): 1500-6.e3.
